# Supplementary material for: Association of High-Intensity Exercise with Renal Medullary Carcinoma in Individuals with Sickle Cell Trait: Clinical Observations and Experimental Animal Studies
Source: Cancers (Basel). 2021 Nov 30;13(23):6022. doi: 10.3390/cancers13236022 (PMC8656882; doi:10.3390/cancers13236022)
Supplement: Supplementary file 1 [file cancers-13-06022-s001.zip › cancers-1457052-supplementary.pdf]

Supplemental Content

Table of Contents

Supplementary Methods Page 2

Epidemiologic comparison Page 2

Mouse strains Page 2

IVIS imaging Page 3

Pimonidazole hydrochloride (Hypoxyprobe) Page 3

Immunohistochemistry Page 3

References Page 5

Supplementary Figures

Supplementary Figure S1 Page 6

Supplementary Figure S2 Page 8

Supplementary Figure S3 Page 11

Supplementary Tables

Supplementary Table S1 Page 12

Supplementary Table S2 Page 13

Supplementary Table S3 Page 14

Supplementary Table S4 Page 15

Supplementary Table S5 Page 16

Supplementary Table S6 Page 17

Supplementary Table S7 Page 18

Supplementary Table S8 Page 19

Supplementary Table S9 Page 20

Supplementary Table S10 Page 21

Supplementary Methods

Epidemiologic comparison

We compared the proportion of military service among our RMC cohort to that of a similarly aged (age 20-40 years old) U.S. population of black individuals with SCT. To estimate this population, we used U.S. census bureau data from 2018 which report that 41,617,764 individuals identify as black (about 12.7% of the U.S. population) [1]. Of this population, about 35% (14,566,217) are between 20 and 40 years old, which is the age most likely to be affected by RMC [1]. Department of defense data from 2018 report that there are 222,705 black individuals serving on active duty age 20 to 40 years old [2]. Sickle cell trait affects about 8% of black individuals [3]; thus, there are about 1,165,297 black individuals age 20 to 40 years with SCT in the U.S., of which a total of 17,816 (1.5%) are on active duty in the U.S. military.

Mouse strains

The Townes model of SCT ($h\alpha/h\alpha::\beta^A/\beta^S$) was generated by Dr. Tim Townes's laboratory and obtained through Jackson Laboratory (Stock No. 013071).[4] The *Cdh16*-Cre strain was generated by Peter Igarashi's laboratory and obtained through Jackson Laboratory (Stock No. 012237). The $Gt(ROSA)26Sor^{tm2(HIF1A/luc)Kael}$ strain was generated by Dr. William G. Kaelin's laboratory and obtained through Jackson Laboratory (Stock No. 006206) [5]. The $Rosa26^{LSL\text{-}TdT}$ was generated by Dr. Hongkui Zeng's laboratory and obtained through the Jackson Laboratory (Stock No: 007908) [6]. Strains were kept in a mixed C57BL/6J and 129Sv/Jae background. All animal studies

and procedures were approved by the UTMDACC Institutional Animal Care and Use Committee.

IVIS imaging

In vivo imaging system (IVIS) was used to detect luciferase activity in mice expressing HIF1α. Mice with pigmented fur were shaved prior to imaging to prevent background caused by melanin in fur. Wild-type mice and mice with SCT were injected with 100 µL of 150 mg/kg of d-luciferin bioluminescence substrate (Perkin Elmer) via intraperitoneal injection (IP) and imaged five minutes after the injection. Living Image 4.3 software was used for analyzing images after image acquisition.

Pimonidazole hydrochloride (Hypoxyprobe)

Renal hypoxia was measured using pimonidazole hydrochloride (Hypoxyprobe). Adult wild-type mice (*N*=52) and mice with SCT (*N* =31) were injected with 100 µL of 60 mg/kg of pimonidazole hydrochloride three hours prior to sacrifice. Mice were then anesthetized using isoflurane (Henry Schein Animal Health), and kidneys were removed and immediately fixed with 10% formalin for 24 hours at room temperature for further IHC studies. The amount of time between removal of the kidneys from mice and fixing in formalin was minimized in order to accurately measure tissue hypoxia.

Immunohistochemistry

IHC was performed on FFPE whole kidney sagittal sections. The sagittal kidney sections were fixed in 10% formalin, embedded, and 5 µm sections were cut using a

microtome (Leica RM2235). The sections were then baked on slides, de-paraffinized, and treated with citrate buffer (Electron Microscopy Sciences) for antigen retrieval according to the manufacturer's instructions. Endogenous peroxidases were then inactivated using 3% hydrogen peroxide (Sigma-Aldrich) for 10 minutes followed by washing with phosphate buffer saline. Non-specific signals were then blocked for 20 minutes using Rodent Block M (Biocare Medical). Samples were then stained with 1:200 primary Hypoxyprobe antibody conjugated with horseradish peroxidase (HRP, rabbit, Hypoxyprobe) for 12 hours overnight in 4°C. After an overnight incubation, slides were washed three times using phosphate buffer saline before staining with Rabbit-on-Rodent HRP polymer (Biocare Medical) for one hour at room temperature. NovaRED peroxidase substrate (Vector Lab) was used for HRP detection (10 minutes of exposure). Hematoxylin was used for counterstaining and eosin was used to detect the cytoplasm of cells in tissue slides. A Nikon EclipseTi microscope and Nikon DS-Fi1 digital camera were used to capture 20x images.

Equal areas of each 20x image were then quantified using ImageJ/FIJI. Using FIJI, the IHC images were deconvoluted into three channels. The optical densities of the DAB channel images were then quantified and graphed into GraphPad for further statistical analysis.

References:

1. U.S. Census bureau (2018). *Race*. retrieved from [https://data.Census.Gov/cedsci/table?Q=race&tid=acsdt1y2018.B02001&hidepreview=false].
2. Department of defense (2018). *2018 demographics report: Profile of the military community.* Retrieved from [https://download.Militaryonesource.Mil/12038/mos/reports/2018-demographics-report.Pdf].
3. Alvarez, O.; Rodriguez, M.M.; Jordan, L.; Sarnaik, S. Renal medullary carcinoma and sickle cell trait: A systematic review. *Pediatr Blood Cancer* **2015**, *62*, 1694-1699.
4. Wu, L.C.; Sun, C.W.; Ryan, T.M.; Pawlik, K.M.; Ren, J.; Townes, T.M. Correction of sickle cell disease by homologous recombination in embryonic stem cells. *Blood* **2006**, *108*.
5. Safran, M.; Kim, W.Y.; O'Connell, F.; Flippin, L.; Günzler, V.; Horner, J.W.; Depinho, R.A.; Kaelin, W.G. Mouse model for noninvasive imaging of hif prolyl hydroxylase activity: Assessment of an oral agent that stimulates erythropoietin production. *Proceedings of the National Academy of Sciences of the United States of America* **2006**, *103*.
6. Madisen, L.; Zwingman, T.A.; Sunkin, S.M.; Oh, S.W.; Zariwala, H.A.; Gu, H.; Ng, L.L.; Palmiter, R.D.; Hawrylycz, M.J.; Jones, A.R., *et al.* A robust and high-throughput cre reporting and characterization system for the whole mouse brain. *Nature neuroscience* **2010**, *13*.

FIGURES

A

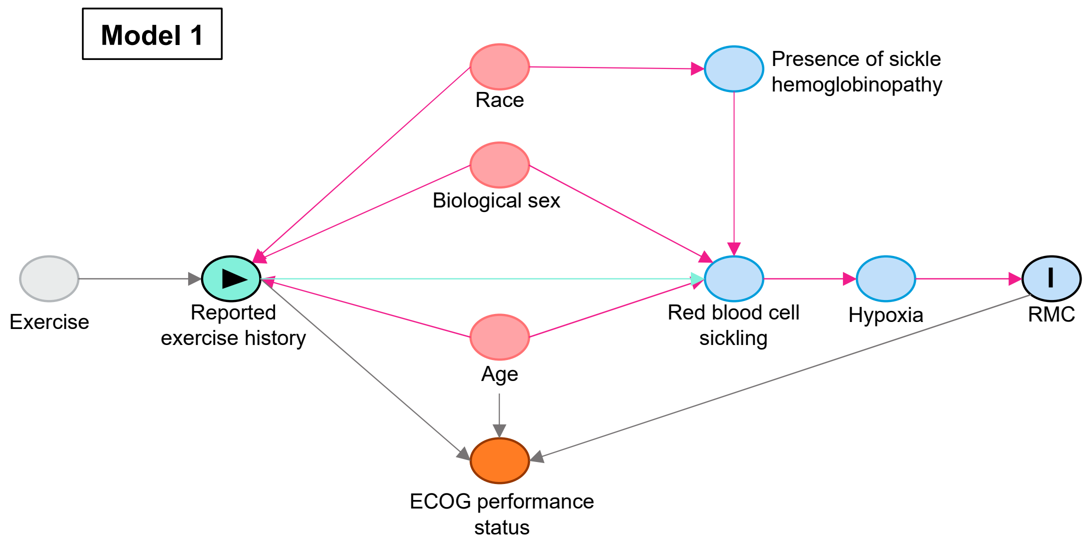

B

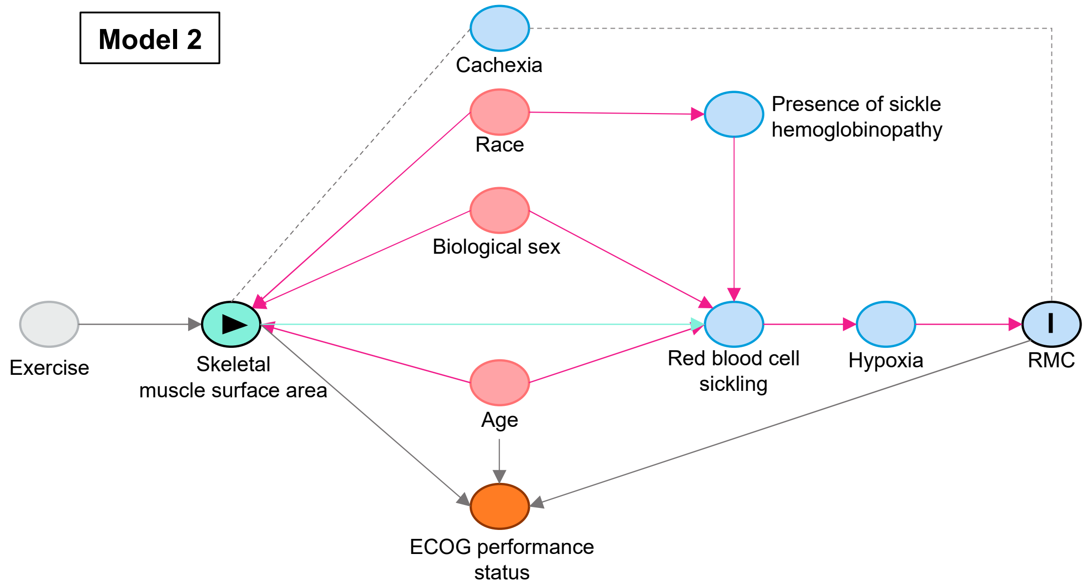

Supplementary Figure S1. Structural causal models. Structural causal models (SCMs) model the causal relationships between exposures and outcomes of interest. Arrows indicate a causal interaction between two variables. Green circles with a triangle represent the exposure of interest and blue circles with **I** represent the outcome of interest (development of RMC). Red circles represent confounding variables that should

be adjusted for in order to determine the causal effect of the exposure on the outcome. Orange circles represent collider variables that can introduce collider bias when included in multivariable regression analyses. Each model was used to construct a multivariable logistic regression adjusting for confounding variables identified in the SCM. **(A)** Model 1 demonstrates the causal relationship between reported exercise history and development of RMC. **(B)** Model 2 demonstrates the causal relationship between standardized skeletal muscle surface area and development of RMC. Given that a relationship may exist between malignancy induced cachexia and skeletal muscle area, we used albumin as a surrogate marker for cachexia and performed an additional multivariable model as a sensitivity analysis including albumin with the confounding variables identified in the SCM.

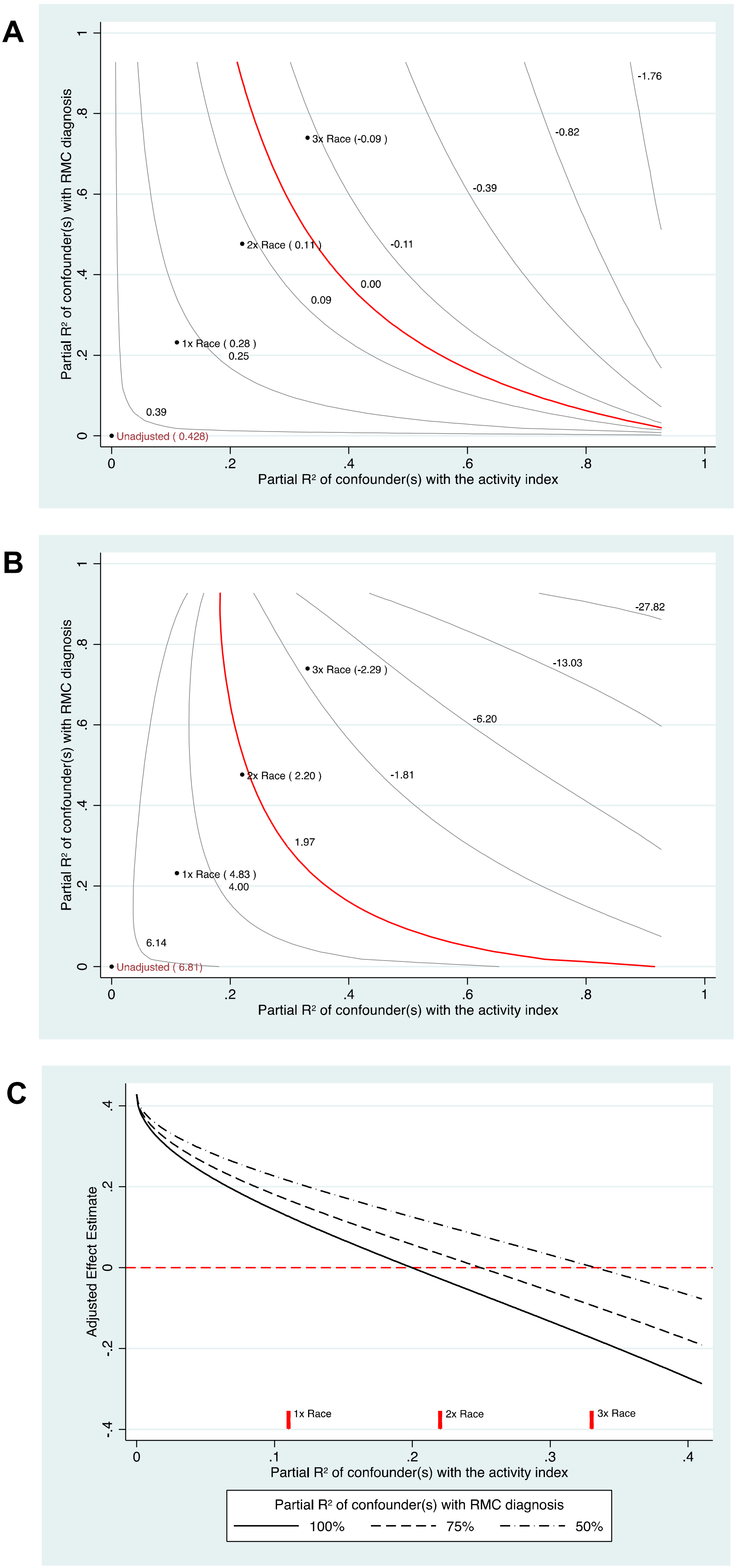
A
Partial R² of confounder(s) with RMC diagnosis
Partial R² of confounder(s) with the activity index
Unadjusted (0.428)
1x Race (0.28)
2x Race (0.11)
3x Race (-0.09)
0.39
0.25
0.09
0.00
-0.11
-0.39
-0.82
-1.76
B
Partial R² of confounder(s) with RMC diagnosis
Partial R² of confounder(s) with the activity index
Unadjusted (6.81)
1x Race (4.83)
2x Race (2.20)
3x Race (-2.29)
6.14
4.00
1.97
-1.81
-6.20
-13.03
-27.82
C
Adjusted Effect Estimate
Partial R² of confounder(s) with the activity index
1x Race
2x Race
3x Race
Partial R² of confounder(s) with RMC diagnosis
100%
75%
50%

Supplementary Figure S2. **Sensitivity plots.** **(A)** Sensitivity contour plot of the point estimate of the activity index coefficient which explores the range of possible estimates that confounders with different strengths could cause. The horizontal axis shows the residual share of variation of the activity index variable that is hypothetically explained by unobserved confounding. The vertical axis shows the hypothetical partial R^2 of unobserved confounding with the outcome of RMC diagnosis. The contours demonstrate what estimate for the activity index would have been obtained in the full regression model including unobserved confounders with such hypothetical strengths. The points on the plot show the bounds on the strength of confounding determined by the covariate "Race". The plot shows that the observed effect of activity index on RMC diagnosis is robust to potential confounding over two times as strong as the observed confounding of the covariate "Race". **(B)** Sensitivity contour plot of the t-value for testing the null hypothesis of zero effect. This plot shows that at a 5% significance level, the null hypothesis of zero association between activity index and RMC diagnosis would still be rejected given confounders up to two times as strong as the covariate "Race". **(C)** Sensitivity plot of extreme scenarios. In this plot, one assumes confounding explains all or a large fraction of the residual variance of RMC diagnosis and varies how strongly such confounding is hypothetically related to the activity index to see how this affects the resulting point estimate. This plot considers confounding that explains 100%, 75% and 50% of the residual variance of the outcome of RMC diagnosis. The red ticks demonstrate the bounds on the strength of the association of a confounder with once, twice or three times the strength of the association of Race with the activity index. The plot demonstrates the extreme case whereby confounding explains 100% of the

residual variance of RMC diagnosis. This extreme case corresponds to the scenario where all our current biological understanding of how RMC pathogenesis is influenced by hypoxia due to red blood cell sickling in the setting of sickle cell trait is completely wrong. In that hypothetical scenario, the putative confounders would have to be nearly two times as strong as "Race" to explain our observations. In the less extreme scenario where the confounding would explain 75% of the residual variance of RMC diagnosis, the putative confounders would have to be over two times as strong as "Race".

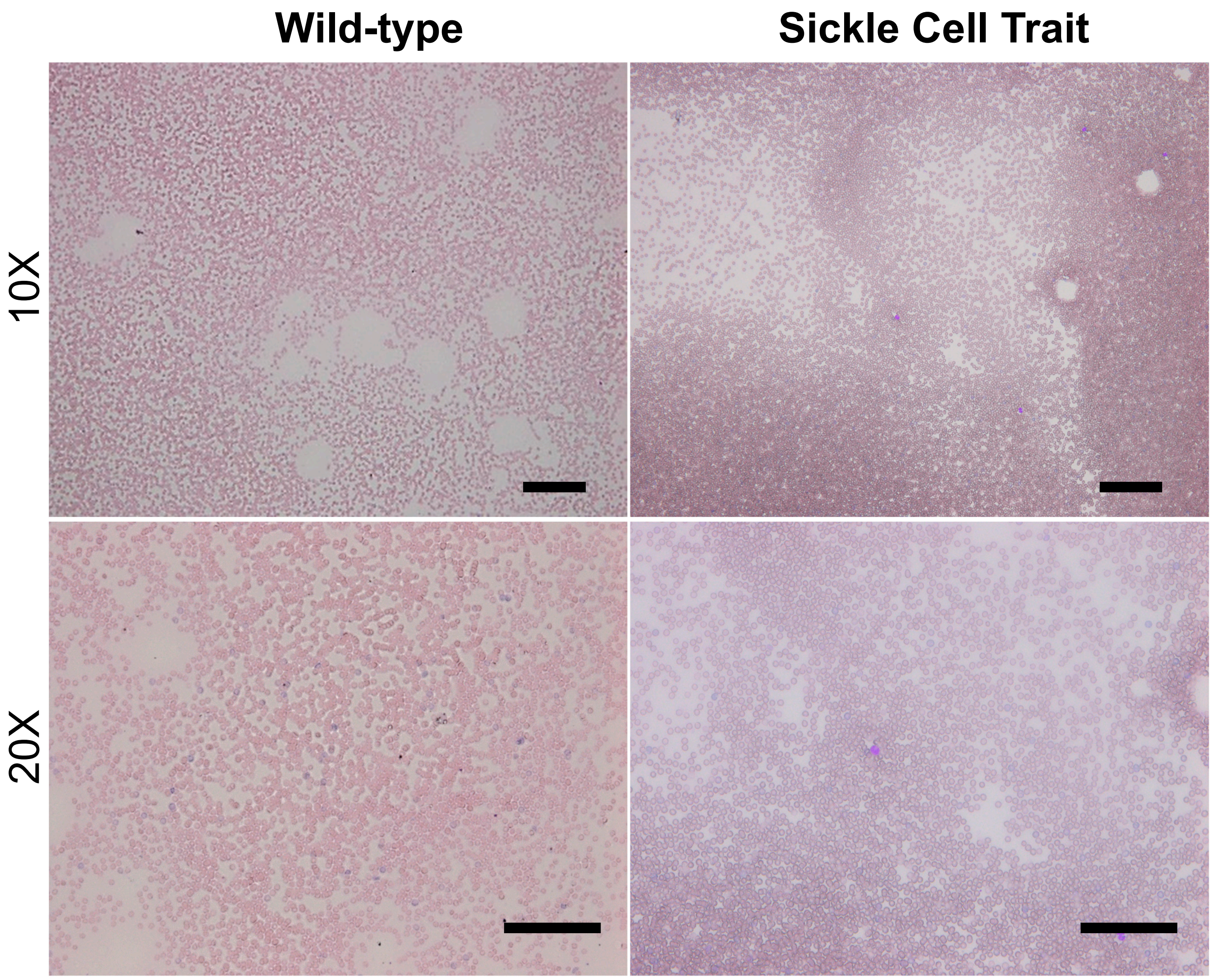

Supplementary Figure S3. Peripheral red blood cell smear comparing wild-type to sickle cell trait mice. Blood was drawn from the tail vein of mice, fixed with methanol, and stained with giemsa. Lack of sickling in peripheral blood is evident in mice with sickle cell trait and comparable to wild-type mice.

Supplementary Table S1. Additional clinical characteristics of patients with RMC.

**Variable**	**RMC (*N*=71)**
Family history RCC, no. (%)	4 (6)
Presenting symptoms, no. (%)	
Pain	55 (78)
Hematuria	39 (55)
Weight loss	19 (27)
Fever	1 (1)
Chills	1 (1)
Night sweats	3 (4)
Localized at diagnosis, no. (%)	8 (11)
Metastatic at diagnosis, no. (%)	63 (89)
T stage, no. (%)	
T1-T2	66 (93)
T3-T4	3 (4)
Unknown	2 (3)
Max tumor diameter, median (IQR), cm	5.8 (5-7)
RMC laterality, no. (%)	
Right kidney	54 (76)
Left kidney	17 (24)
Multiple metastatic locations, no. (%)	55 (78)
Nephrectomy, no. (%)	50 (70)

Supplementary Table S2. Metastatic locations among patients with RMC and metastatic disease at initial diagnosis.

**Metastatic site**	***N*=63 (%)**
Retroperitoneal nodes	52 (83)
Lungs	45 (71)
Bone	15 (24)
Liver	14 (22)
Hilar LN	13 (21)
Soft tissue	5 (8)
Supraclavicular LN	4 (6)
Ipsilateral adrenal	4 (6)
Pancreas	2 (3)
Thyroid	2 (3)
Mesentery	1 (2)
Ovary	1 (2)
Spleen	1 (2)
Brain	1 (2)
Bowel	0
Contralateral kidney	0
Contralateral adrenal	0

Supplementary Table S3. Cancer type and stage for matched control patients.

**Matched Patient Cancer Type**	***N* = 122 (%)**	**Stage III, *N* (%)**	**Stage IV, *N* (%)**
Testicular cancer	46 (38)	46 (100)	0
Renal cell carcinoma	46 (38)	13 (28)	33 (72)
Bladder cancer	21 (17)	4 (19)	17 (81)
Prostate cancer	9 (7)	0	9 (100)

Supplementary Table S4. Full multivariable model evaluating association between physical activity index and diagnosis of RMC.

Variable	Odds Ratio	95% CI	*P* value
Physical activity index	10.4	4.5-23.9	<0.001
Age	1.01	0.97-1.05	0.7
Gender			
Male	Ref		
Female	1.6	0.7-3.6	0.3
Race			
Black	Ref		
Caucasian	0.09	0.04-0.2	<0.001
Hispanic	0.97	0.2-5.9	0.98
Asian	0.2	0.02-2.6	0.2

Supplementary Table S5. Full multivariable model evaluating association between standardized skeletal muscle surface area and diagnosis of RMC.

**Variable**	**Odds Ratio**	**95% CI**	***P* value**
Skeletal muscle surface area	1.04	1.00-1.08	0.03
Age	0.98	0.95-1.02	0.4
Gender			
Male	Ref		
Female	2.2	0.9-5.7	0.1
Race			
Black	Ref		
Caucasian	0.08	0.03-0.2	<0.001
Hispanic	0.4	0.7-2.6	0.4
Asian	0.3	0.03-2.4	0.2

Supplementary Table S6. Multivariable model including albumin evaluating the association between standardized skeletal muscle surface area and diagnosis of RMC.

Variable	Odds Ratio	95% CI	*P* value
Skeletal muscle surface area	1.04	1.00-1.08	0.04
Age	0.99	0.95-1.02	0.5
Gender			
Male	Ref		
Female	1.9	0.7-5.1	0.2
Race			
Black	Ref		
Caucasian	0.08	0.03-0.2	<0.001
Hispanic	0.4	0.06-2.2	0.3
Asian	0.3	0.03-2.5	0.2
Albumin	0.4	0.2-0.98	0.045

Supplementary Table S7. Multivariable model including smoking evaluating the association between physical activity index and diagnosis of RMC.

Variable	Odds Ratio	95% CI	*P* value
Physical activity index	9.6	4.1-22.3	<0.001
Age	1.01	0.97-1.1	0.5
Gender			
Male	Ref		
Female	1.28	0.55-3.0	0.6
Race			
Black	Ref		
Caucasian	0.08	0.03-0.2	<0.001
Hispanic	1.4	0.2-9.0	0.7
Asian	0.2	0.01-1.7	0.1
Smoking	0.3	0.1-0.8	0.01

Supplementary Table S8. Multivariable model including smoking evaluating the association between standardized skeletal muscle surface area and diagnosis of RMC.

Variable	Odds Ratio	95% CI	*P* value
Skeletal muscle surface area	1.04	1.00-1.08	0.03
Age	0.99	0.95-1.02	0.7
Gender			
Male	Ref		
Female	1.9	0.2-0.69	0.2
Race			
Black	Ref		
Caucasian	0.08	0.03-0.2	<0.001
Hispanic	0.7	0.1-4.3	0.7
Asian	0.2	0.02-1.6	0.1
Smoking	0.3	0.1-0.7	0.004

Supplementary Table S9. Clinical characteristics of prospectively evaluated patients with RMC.

**Variable**	*N*=7
Age, median (IQR)	28 (17-43)
Biological sex, no. (%)	
Male	4 (57)
Female	3 (43)
Race, no. (%)	
African American	7 (100)
Sickle hemoglobinopathy, no. (%)	
Sickle cell trait	6 (86)
Sickle beta-thalassemia	1 (14)
Comorbidities	
HTN	5 (71)
Other	4 (57)
ECOG, no. (%)	
0	0 (0)
1	5 (71)
2	2 (29)
BMI, median (IQR), kg/m^2	20.1 (18.6-28)
Albumin, median (IQR), g/dL	4.2 (3.8-4.5)
Smoking history, no. (%)	2 (29)
Exercise history, no. (%)	5 (71)
Athlete, no. (%)	5 (71)
Military, no. (%)	0 (0)
Activity index, no. (%)	5 (71)
RMC laterality, no. (%)	
Right kidney	3 (43)
Left kidney	4 (57)

Supplementary Table S10. Prospectively collected exercise history of seven patients with RMC.

ID	Exercise intensity	Exercise time per week	Participation prior to RMC diagnosis	Exercise Description	Primary tumor laterality
1	High	≥3 hours	>3 years	Softball and swimming	Left
2	High	≥2 hours	>3 years	Basketball and frequent gym exercise	Right
3	High	≥2 hours	>3 years	Basketball, soccer, weightlifting	Right
4	High	≥3 hours	>3 years	Professional athlete	Left
5	None	None	None	Sedentary lifestyle	Left
6	High	≥3 hours	>3 years	Track, martial arts, and frequent gym exercise	Right
7	None	None	None	Sedentary lifestyle	Left
